# Biological effect of ketamine in urothelial cell lines and global gene expression analysis in the bladders of ketamine-injected mice

**DOI:** 10.3892/mmr.2014.2823

**Published:** 2014-10-30

**Authors:** CHENG-HUANG SHEN, SHOU-TSUNG WANG, YING-RAY LEE, SHIAU-YUAN LIU, YI-ZHEN LI, JIANN-DER WU, YI-JU CHEN, YI-WEN LIU

**Affiliations:** 1 Department of Urology, Chiayi Christian Hospital, Chiayi Christian Hospital, Chiayi 600, Taiwan, R.O.C.; 2 Department of Microbiology, Immunology and Biopharmaceuticals, College of Life Sciences, Chiayi Christian Hospital, Chiayi 600, Taiwan, R.O.C.; 3 Department of Food Science, National Chiayi University, Chiayi Christian Hospital, Chiayi 600, Taiwan, R.O.C.; 4 Department of Medical Research, Chiayi Christian Hospital, Chiayi 600, Taiwan, R.O.C.; 5 Department of Nursing, Min-Hwei College of Health Care Management, Tainan 73658, Taiwan, R.O.C.; 6 Department of Pathology, Chiayi Christian Hospital, Chiayi 600, Taiwan, R.O.C.

**Keywords:** bladder, cytotoxicity, gene expression profiling, ketamine, keratin

## Abstract

Ketamine is used clinically for anesthesia but is also abused as a recreational drug. Previously, it has been established that ketamine-induced bladder interstitial cystitis is a common syndrome in ketamine-abusing individuals. As the mechanisms underlying ketamine-induced cystitis have yet to be revealed, the present study investigated the effect of ketamine on human urothelial cell lines and utilized a ketamine-injected mouse model to identify ketamine-induced changes in gene expression in mice bladders. In the *in vitro* bladder cell line assay, ketamine induced cytotoxicity in a dose- and time-dependent manner. Ketamine arrested the cells in G1 phase and increased the sub-G1 population, and also increased the barrier permeability of these cell lines. In the ketamine-injected mouse model, ketamine did not change the body weight and bladder histology of the animals at the dose of 30 mg/kg/day for 60 days. Global gene expression analysis of the animals’ bladders following data screening identified ten upregulated genes and 36 downregulated genes induced by ketamine. A total of 52% of keratin family genes were downregulated, particularly keratin 6a, 13 and 14, which was confirmed by polymerase chain reaction analysis. Keratin 14 protein, one of the 36 ketamine-induced downregulated genes, was also reduced in the ketamine-treated mouse bladder, as determined by immunohistochemical analysis. This suggested that cytotoxicity and keratin gene downregulation may have a critical role in ketamine-induced cystitis.

## Introduction

Ketamine was first synthesized in 1962 (1) and introduced into clinical medicine for dissociative anesthesia in 1970 (2). It is a non-competitive *N*-methyl-d-aspartic acid receptor antagonist and used as a short-acting general anesthetic in human and veterinary clinical settings (3). Due to its low cost and the fact that it induces hallucination and alters the state of consciousness, ketamine soon emerged as a recreational drug (4). As the number of ketamine abusers gradually increased, a new side effect on the bladder was first reported in 2007 (5,6). The symptoms of ketamine-induced cystitis include dysuria and urgency (7,8), and cystoscopic examination of severe cases demonstrated hemorrhagic cystitis, denuded mucosa and marked inflammation (9). The symptoms are similar to interstitial cystitis (IC) (6), therefore, certain treatment regimens, including oral pentosan polysulphate and intravesical instillation of hyaluronic acid, are used in ketamine-induced cystitis, to relieve the varying degrees of symptoms (6,9,10). Other medicines, including antibiotics, non-steroidel anti-inflammatory drugs, steroid and anticholinergic drugs, are also applied for therapy, but the benefit is limited (9).

To date, no specific treatment for patients with ketamine-induced cystitis has been established. In spite of the increase in the number of recreational users, investigating ketamine-induced cystitis in humans is not simple. As a result, a number of studies have used animal models for investigating the mechanisms of action and effects of ketamine. Previously, to the best of our knowledge, two mouse model (11,12) and two rat model (13,14) *in vivo* studies have been published. In Yeung *et al*’s mouse model (11), 30 mg/kg/day ketamine injection induced submucosal infiltration of mononuclear inflammatory cells. The urothelium became thinner and the number of nerve fibers was reduced following one month of ketamine treatment. In Meng’s mouse study (12), following 100 mg/kg/day ketamine injection for 2~4 months, a decrease in the mouse body weight growth rate and bladder capacity, the increase of adenosine triphosphate-evoked detrusor contraction and P2X1 receptor protein was observed in the animal bladders. In Gu’s rat model (13), the whole rat bladder proteins were analyzed by two-dimensional electrophoresis following 50 mg/kg/day ketamine injection for four months. The bladder histological examination demonstrated hyperplastic urotheliums and inflammatory cell infiltration. The phosphorylated transgelin of bladder smooth muscle was increased by ketamine treatment, which suggested that transgelin may have a role in decreasing bladder contractility. In Chuang’s rat study (14), it was revealed that 25 mg/kg/day ketamine injection for one month induced cyclooxygenase-2 and inducible nitric oxide synthase gene expression in the rat bladders.

Although the animal studies mentioned above provided notable insight into the mechanisms of ketamine-induced bladder damage, these effects remain to be fully elucidated. In the present study, three urothelial cell lines were used to study the cytotoxicity of ketamine and the barrier permeability affected by ketamine. In the *in vivo* assay, a mouse animal model was designed for global gene expression analysis in the bladders.

## Materials and methods

### Cell culture and ketamine treatment

Three different urothelial cell lines, purchased from Bioresource Collection and Research Center (Hsinchu, Taiwan) were used. The SV-HCU-1 cell line derived from normal human urothelial cells immortalized by the SV40 virus. The RT4 cell line is derived from a well-differentiated papillary tumor of the human bladder (15). The 5637 cell line is a grade II carcinoma of the human bladder (16). SV-HUC-1 cells were cultured in Ham’s F-12 medium (Gibco Life Technologies, Grand Island, NY. USA) supplied with 7% fetal bovine serum (FBS; Biological Industries, M.P. Ashrat, Israel). RT4 cells were cultured in McCoy’s 5A medium (Sigma-Aldrich, St. Louis, MO, USA) supplied with 10% FBS, 1% penicillin and 1% streptomycin. 5637 cells were maintained in RPMI-1640 medium (Gibco Life Technologies) supplied with 10% FBS, 1% penicillin and 1% streptomycin (Gibco Life Technologies). The cells were incubated in a CO_2_ incubator at 37°C, with 5% CO_2_ and 95% filtered air. Ketamine (Sigma-Aldrich) was dissolved in normal saline. For the cultured cell assay, ketamine was added to cells of the ketamine-treated groups, while the same volume of normal saline was added to the control cells.

### Cell viability assay

The cell number was determined by a colorimetric MTT assay. The cells were seeded in 96-well plates for 24 h, then were incubated with various concentrations of ketamine or normal saline for another 24–48 h. MTT was added into the medium for 2 h, then the medium was discarded and dimethyl sulfoxide was added to dissolve the formazan product. Each well was measured by light absorbance at 490 nm. The result was expressed as the percentage of the normal saline-treated control group.

### Cell cycle analysis

The cells were seeded in 100-mm dishes. Following 24 h incubation, ketamine or normal saline was added. Following treatment for 24 and 48 h, the cells were trypsinized, centrifuged at 800 × g for 5 min and fixed with ice-cold 75% ethanol overnight at 4°C. Following removal of the ethanol, the cells were stained with a DNA staining solution [containing 1 mg/ml propidium iodide and 10 mg/ml RNase A dissolved in phosphate-buffered saline (PBS)] for 30 min at room temperature. The DNA content of the stained cells was measured using a FACScan flow cytometer. The cell doublets were removed by gating the left area of the FL2-W/FL2-A plot for analysis. The cell cycle data from flow cytometry were analyzed using ModFit LT^TM^ software (Verity Inc. Sunnyvale, CA).

### Urothelial barrier function assay

Approximately 4×10^4^ SV-HUC-1 cells, 4×10^4^ RT4 cells and 1×10^4^ 5637 cells were seeded on an Transwell insert with 0.4 μm pore size filter membrane (Millipore Corp. Billerica, MA, USA) and incubated for 24 h. Ketamine was added into the upper and lower chamber media at the same time. Following incubation for 19 or 43 h, green fluorescence-labeled antibodies (Alexa Fluor^®^ 488 goat anti-mouse immunoglobulin G; Invitrogen Life Technologies) were added into upper chamber medium (9.6 μg/insert) and continued incubating for another 5 h. The total medium in the upper and lower chambers were collected for fluorescence analysis by a fluorescence microplate reader (excitation/emission: 488/519 nm).

### Animals and ketamine treatment

Six-week-old male Balb/c mice were used in the present study and purchased from the National Laboratory Animal Center (Taipei, Taiwan). All of the animals were maintained at the qualified animal care facility of Biotechnology and Health Hall in National Chiayi University (Chiayi City, Taiwan, R.O.C) for one week prior to intraperitoneal (i.p.) injection. At seven weeks of age, the mice were divided into four groups (12 mice/group), including control-30 days (i.p. normal saline for 30 days), ketamine-30 days (i.p. 30 mg/kg/day ketamine for 30 days), control-60 days (i.p. normal saline for 60 days) and ketamine-60 days (i.p. 30 mg/kg day ketamine for 60 days). The mice were housed in polycarbonate cages, provided with food and water ad libitum and maintained on a 12 h light-dark cycle at 22±2°C. All of the experiments were approved by the Institutional Animal Care and Use Committee of National Chiayi University.

### Bladder tissue collection and hematoxylin and eosin staining

Following the 30- or 60-day treatment, the mice were euthanized and the bladder tissues were removed. A total of 20 bladders (five/group) were fixed in 10% neutral formalin for histological examination, three bladders/group were homogenized together and RNA was extracted, and the other bladders were stored under liquid nitrogen for future use. The bladder tissues in 10% neutral formalin were embedded in paraffin and then cut into 4-μm sections on glass slides. One slide from each mouse was stained with hematoxylin and eosin (H&E). Other slides were prepared for immunohistochemical analysis.

### Global gene expression analysis

Total RNA was isolated from three bladders in each group using TRIzol reagent (Invitrogen Life Technologies) according to the manufacturer’s instructions. The quality of RNA was examined using Agilent’s RNA LabChip kits on the 2100 Bioanalyzer (Agilent Technologies, Inc., Santa Clara, CA, USA). The RNA samples from the four groups (control-30 days, ketamine-30 days, control-60 days and ketamine-60 days) were transferred to fluorescence-labeled antisense (a)RNA using OneArray Amino Allyl aRNA Amplification kit (Phalanx Biotech Group, Hsinchu, Taiwan) and Cy5 dye labeling (Amersham Pharmacia, Piscataway, NJ, USA). For global gene expression analysis, the fluorescent targets were hybridized to the Mouse Whole Genome OneArray^TM^ version MOA 2.0 (Phalanx Biotech Group), containing 27,295 mouse genome probes. One mixture sample was applied to two chips, and the normalized intensities were calculated from raw intensities by median scaling. Microarray image scanning and data analysis were achieved by Phalanx Biotech Group.

### Polymerase chain reaction (PCR) analysis

Reverse transcription was performed on 2 μg of total RNA by 5 μM random hexamer and RevertAid^TM^ reverse transcriptase (Thermo Fisher Scientific, Fermentas, Pittsburgh, PA, USA), then 1/10 volume of reaction mixture was used for PCR with specific primers (keratin 6a forward 5′-TGCCAGGGGCAAGCTGGAAG-3′ and reverse 5′-ACGGGATTCTGCAGCCATGACA-3′; keratin 13 forward 5′-AGCTTGGAGGAGGCCGTAAT-3′ and reverse 5′-AAGCACTGTAGTCCCGCTCT-3′; keratin 14 forward 5′-TGGTGCAGAGCGGCAAGAGTG-3′ and reverse 5′-TGCGGATCTGGCGGTTGGTGG-3′) and β-actin forward 5′-CCTAAGGCCAACCGTGAAAAG-3′ and reverse 5′-TCTTCATGGTGCTAGGAGCCA-3′). The PCR products (keratin 6a, 486 bp; keratin 13, 375 bp; keratin 14, 399 bp; β-actin, 623 bp) were analyzed by 1% agarose gel.

### Immunohistochemical analysis

After being washed in PBS, the slides were incubated in a blocking solution for 30 min and then with primary antibodies against keratin 14 (Genetex, Taipei, Taiwan) at a 1:100 dilution at 4°C overnight. The slides were then washed and incubated with secondary antibodies containing horseradish peroxidase at 25°C for 30 min. Following this treatment, the slides were washed with PBS and further incubated with 3,3′-diaminobenzidine for 5 min. Finally, the sections were rinsed in running water, treated with hematoxylin for ~10–15 sec and mounted for evaluation.

### Statistical analysis

Numerical data (except gene expression microarray data) are expressed as the mean ± standard error. Statistical differences were analyzed by one-way analysis of variance analysis of variance followed by Tukey’s test. All statistics were calculated using SigmaState version 3.5 (Systat Software, San Jose, CA, USA)

## Results

### Cytotoxicity of ketamine in human urothelial cell lines SV-HUC-1, RT4 and 5637

**Figure 1:**
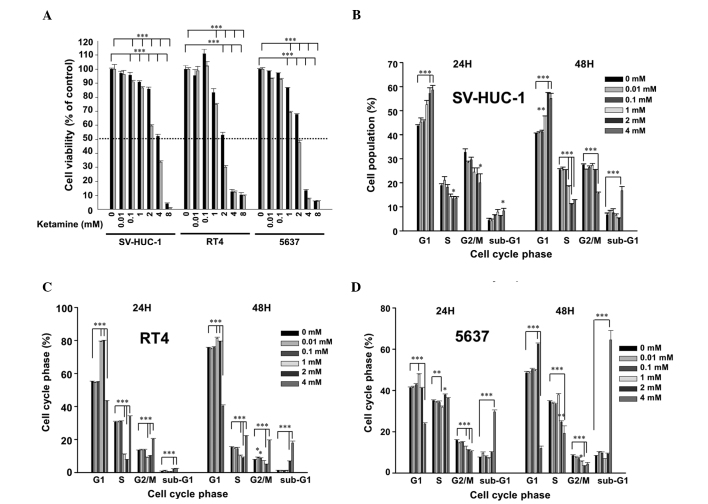
Cytotoxicity of ketamine on SV-HUC1, RT4 and 5637 cells. (A) Cytotoxicity of 0–8 mM ketamine. The cells were treated with 0–8 mM ketamine for 24 h (black bar) and 48 h (gray bar), and the cell viability was analyzed by an MTT assay. (B–D) Cell cycle distribution changed following incubation with 0–4 mM ketamine for 24 and 48 h. The cells were collected for cell cycle analysis following ketamine treatment in (B) SV-HUC1, (C) RT4 and (D) 5637 cells. Quantification performed from three independent experiments. ^*^P<0.05, ^**^P<0.01, ^***^P<0.001, significant difference between the control and ketamine-treated cells.

Following ketamine treatment for 24 h, the IC_50_ value of ketamine was ~4, 2 and 3 mM in SV-HUC-1, RT4 and 5637 cells, respectively. At 48 h, the IC_50_ was ~3, 1.5 and 2 mM in the SV-HUC-1, RT4 and 5637 cells, respectively (Fig. 1A). These results suggested that ketamine is cytotoxic to urotheliums in a dose-dependent and time-dependent manner. Due to the identified cytotoxicity, ketamine-induced cell cycle changes were analyzed. In the SV-HUC-1 cells, ketamine dose-dependently increased the G1 phase cells at a dose higher than 1 mM and significantly increased the sub-G1 level at 4 mM (Fig. 1B). In the RT4 (Fig. 1C) and 5637 (Fig. 1D) cells, ketamine also arrested the cells in the G1 phase between 1 to 2 mM, and significantly increased the sub-G1 level at 4 mM. All of the above data suggested that ketamine induced G1 arrest and cytotoxicity in the human urothelial cells.

### Ketamine increases barrier permeability of human urothelial cells

**Figure 2:**
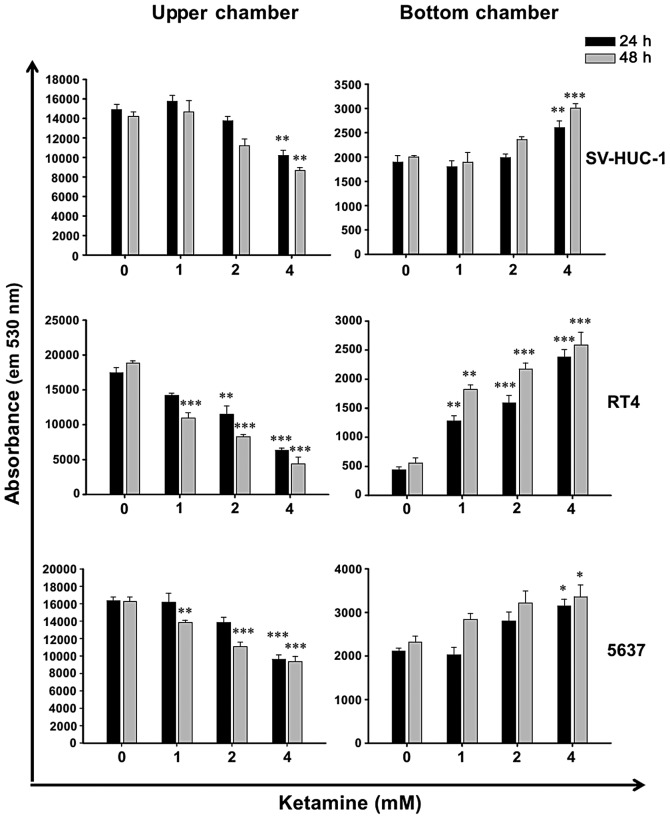
Ketamine increases urothelium barrier permeability in SV-HUC1, RT4 and 5637 cells. The cells were treated with 0–4 mM ketamine for 24 or 48 h, then the upper and bottom chamber media were incubated with Alexa Fluor^®^ 488 goat anti-mouse immunoglobulin G and analyzed by a fluorescence microplate reader. Data are presented as the mean ± standard deviation of three independent experiments. ^*^P<0.05, ^**^P<0.01, ^***^P<0.001, significant difference between the control and ketamine-treated cells.

Due to the cytotoxicity of ketamine (Fig. 1), it was hypothesized that ketamine may decrease epithelial barrier function. Therefore, the urothelial barrier permeability assay was employed. Following ketamine treatment for 24 and 48 h, the permeability of green fluorescence-labeled antibodies was increased dose-dependently in SV-HUC-1, RT4 and 5637 cells (Fig. 2). When comparing the cytotoxicity of ketamine and its enhancing effect on the barrier permeability, it was evident that the dose causing cytotoxicity accompanied barrier function loss. This suggested that the cytotoxic effect of ketamine may, at least in part, cause the loss of barrier function in ketamine-treated urotheliums.

### Effect of daily ketamine injection on mouse body weight, behavior and bladder tissue histology

In addition to the *in vitro* assay, the present study aimed identify the gene expression in ketamine-treated mouse bladder. Following daily ketamine injection for 30 and 60 days, the growth rate of murine body weight was not significantly different between the control and ketamine-treated group (data not shown). This suggested that intraperitoneal administration of 30 mg/kg/day ketamine for 60 days may not affect the physiological properties of the mice. At this dosage, that the mice displayed symptoms of excitation following ketamine injection for 2–5 min, which lasted for ~40 min. During the injection period, the onset of excitation was gradually delayed and its intensity was also gradually decreased. This suggested that the mice developed a tolerance to ketamine-induced excitation. At the 30th and 60th day, the bladders were isolated for tissue examination. The histology of bladder tissues demonstrated no evident differences between the control and ketamine groups at 30 and 60 days of treatment (data not shown).

### Global gene expression analysis in the bladders of ketamine-injected mice

Gene expression microarray analysis of bladder tissue was applied to compare gene expression between the control and ketamine-treated animals. Upregulated genes with differential expression (fold change log 2 ≥ 1 and P<0.05) at 60 days and a statistical difference (only P<0.05) at 30 days were selected. Downregulated genes with differential expression (fold change log 2 ≤ −1 and P<0.05) at 60 days and statistical difference (only P<0.05) at 30 days were selected. Analysis revealed that 10 genes were upregulated (Table I) and 36 genes were downregulated (Table II only reveals the top ten genes and keratin 78). Among these 46 genes, two keratin genes which were associated with cell-cell/basement membrane adhesion function were found to be significantly decreased. Of note, the amount of type I keratin was also decreased in the ketamine-treated rat bladders in the study by Gu *et al* (13).

### Keratin 14 gene expression is decreased in ketamine-treated mouse bladders

**Figure 3:**
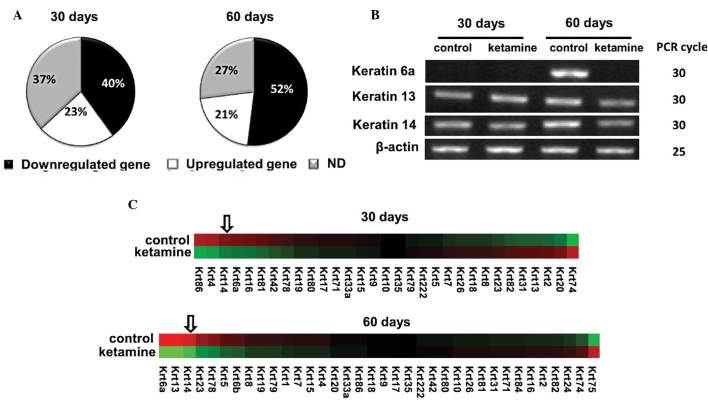
Comparison of keratin family gene expression in the control and ketamine-treated mouse bladders. (A) The pie chart of keratin family genes reveals the percentage of downregulated, upregulated and ND keratin genes. (B) Polymerase chain reaction analysis of three keratin gene expression. The RNA was extracted from mouse bladders following normal saline (control) or 30 mg/kg/day ketamine (ketamine) i.p. injection for 30 and 60 days. The product sizes of keratin 6a, 13, 14 and β-actin are 486, 375, 399 and 623 bp, respectively. (C) Heat map of keratin gene expression. The map was created using the Cluster 3.0. The genes are arrayed from the most downregulated (left) to the most upregulated (right).

**Figure 4:**
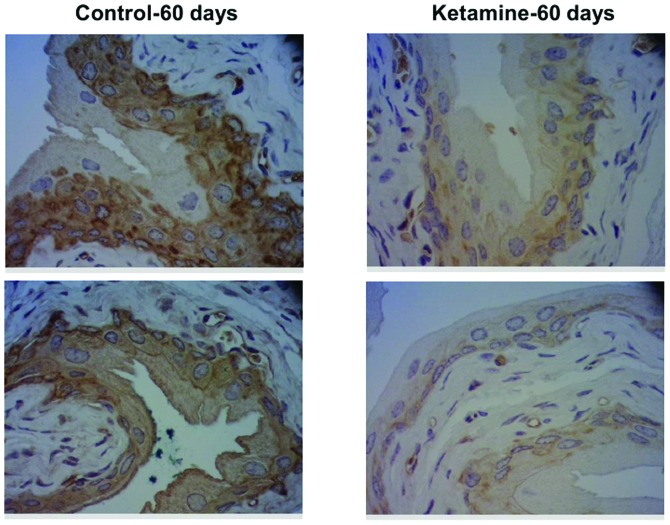
Keratin 14 protein expression in mouse bladder tissues by immunohistochemical analysis. The slides of bladder tissue were hybridized with anti-keratin 14 antibodies and then photographed under ×400 microscopy. Upper and lower images, representative images from two different mouse bladders.

Cytoskeletal keratins belong to intracellular intermediate filaments that connect to epithelial cell adhesion plaques in macula adherens and hemidesmosome sites. Numerous inherited skin-blistering diseases are caused by keratin gene mutations. There were 52 keratin family genes in the gene expression microarray chip. The majority of the keratins were downregulated by ketamine: 40% following 30 days and 52% following 60 days (Fig. 3A). The top ten downregulated keratins in the 60-day treatment are listed in Table III. The top three downregulated keratins, including 6a, 13 and 14 were confirmed by PCR analysis (Fig. 3B). Following deleting the genes with no significant difference (P>0.05), a heat map of residue keratin genes was constructed (Fig. 3C). Among the downregulated keratin genes, keratin 14 gene was among the top three genes following 30- and 60-day treatment. Keratin 14 was also among the selected top ten downregulated genes in Table II. To confirm the protein expression change of keratin 14, immunohistochemical analysis was applied. The results demonstrated that keratin 14 protein expression was also decreased in the 60-day murine urothelium (Fig. 4).

## Discussion

In the present *in vitro* study, it was identified that ketamine damaged urotheliums and decreased barrier function in a dose-dependent manner. In the *in vivo* mouse study, it was demonstrated that ketamine decreased the expression of numerous keratin genes, including keratin 14. Keratin 14 protein is also decreased in ketamine-treated mouse bladders. This suggested that cytotoxicity may cause the loss of urothelial barrier function at high doses of ketamine, while at low doses, keratin gene downregulation may be a sign of urothelial disorder.

Ketamine demonstrated toxicity (Fig. 1A) and induced sub-G1 formation in a dose- and time-dependent manner (Fig. 1B–D). Therefore, it may be concluded that highly frequent and repeated doses of ketamine may eventually cause urothelial damage in abusive, recreational users. The cytotoxicity of ketamine has been reported in neuroblastoma (17,18), lymphoma Jurkat cells (18) and hepatoma (19). In these three studies, it was collectively suggested that ketamine induced cell death via apoptosis and urothelial apoptosis was also found in abusers (20). In addition, the present study also analyzed the barrier function of urotheliums *in vitro*. The results indicated that ketamine also increased barrier permeability in a dose-dependent manner (Fig. 2).

According to Yeung’s ICR mouse model (30 mg/kg/day for 1 and 3 months) (11), ketamine injection induces urothelial degeneration and inflammatory cell infiltration in bladders. In the present study, the same dose of ketamine was used in Balb/c mice, and no inflammatory phenomenon was observed by histological examination. Four inflammatory genes (cyclooxygenase-2, nitric oxide synthase-2, interleukin-6 and -10) from microarray data were selected for analysis by PCR and no visual PCR products were observed on the gel (data not shown). This result suggested that inflammation had not yet occurred in the Balb/c mouse bladder tissue following 30 mg/kg/day ketamine treatment for 30 and 60 days. These data were consistent with the result of the histological analysis, using H&E staining, which demonstrated no inflammatory cell infiltration in the bladder tissue. In Meng’s mouse model (100 mg/kg/day for 1 to 4 months) (12), another mouse strain C57BL/6 was used with higher ketamine dosages. The authors identified that ketamine reduced the mouse weight growth and induced micturition following eight weeks. The bladder histology also demonstrated urothelial degeneration and mononuclear cell infiltration, while submucosal congestion was not present in Yeung’s results. These results suggested that the strain and ketamine dose may affect the level of ketamine-induced bladder disorder. In addition to strain and ketamine dosage, gender may be another reason for differences at the damage level. The same strain of Sprague-Dawley rats but a different gender was used in Gu *et al’*s study (50 mg/kg/day for 16 weeks, male rats) (13) and Chaung *et al’*s study (25 mg/kg/day for four weeks, female rats) (14). According to Gu *et al’*s study, ketamine increased the urinary frequency and induced hematuria, hyperplastic epithelium and inflammatory cell infiltration in the bladder (13). By contrast, according to Chaung *et al’*s study, using a lower ketamine dose and short treatment time, ketamine induced urothelial degeneration, red blood cell debris accumulation in bladder cavity and mononuclear cell infiltration. The urothelial mucosal damage of female rats appeared to be more severe than that of male rats.

Keratins are the major component of the fibrous intermediate filament in epithelial cells. Keratin 20 is a tumor marker of urothelial dysplasia (21). One study found that keratin 20 expression decreased in the bladder of ketamine-abusing individuals (22). The microarray data of the present study also indicated that keratin 20 decreased within 60 days (Table IV). Different keratins are expressed in different layers of the urothelium (basal, intermediate and umbrella), keratin 20 is in the umbrella layer and keratin 14 is in the basal/intermediate layers (23). Keratin 14 and 5, type I and type II keratins, assemble to heterodimers, and thousands of them assemble to 10-nm-wide intermediate filament cytoskeleton. According to the present study, keratin 5 was also decreased in the bladders of ketamine-treated mice. Mutations in keratin 14 or keratin 5 cause a rare genetic disease called epidermolysis bullosa simplex (24). In addition to the representative skin bullous lesions, patients with epidermolysis bullosa simplex also demonstrated fragility of epithelial tissues in the genitourinary tract, which caused voiding difficulty and urinary retention (25). The cell proliferation rate was reduced following knockdown of keratin 14 (26). It remains elusive whether or not the downregulation of keratin genes induces urinary disorders in ketamine abusers, and therefore, it is worthy of further study.

In addition to decreases in the levels of various types of keratin, the cDNA array data indicated further mechanisms leading to the downregulation of urothelial barrier function. Firstly, the hemidesmosome, consisting of intracellular keratins, plectin plaque and adhesion molecules, such as the α6β4 integrin (27), contributed to the firm attachment between urothelium and extracellular matrix. In the array data, integrin α6/β4 demonstrated a marked decrease as well at 60 days (Table IV), which may implicate the hemidesmosome was collapsing and cell is progressively denuding from basal lamina. Secondly, it was identified the claudin-1 expression was also downregulated at 60 days (Table IV). This suggested that tight junctions of the urothelium may also have been affected. Although bladder damages were not identified in the 30 mg/kg/day ketamine-treated Balb/c mice, the microarray data demonstrated certain molecular defects which correlated with urothelial barrier function. Additional studies are required to further elucidate these correlations.

## Figures and Tables

**Table I:** Upregulated genes with differential expression (fold-change log 2 ≥ 1 and P<0.05) at 60-day ketamine treatment and statistical difference (P<0.05) at 30-day ketamine treatment in mouse bladders.

		Normalized intensity				Ratio of change (%)	
		
		30-day		60-day		(K-C)/C × 100%	
		
Gene name	Accession number	C	K	C	K	30-day	60-day
Hedgehog-interacting protein	NM_020259.4	296.8	602.8	183.8	847.5	103.1	361.1
Fucosyl-transferase 9	NM_010243.3	165.3	226.6	78.2	330.7	37.1	322.9
Leucine rich repeat containing G protein coupled receptor 5	NM_010195.2	97.3	143.4	149.1	435.0	47.4	191.8
Titin-cap	NM_011540.2	264.0	501.2	282.9	741.5	89.8	162
Family with sequence similarity 55, member C	NM_001134494.1	438.0	705.3	170.4	401.8	61.0	135.8
Toll-like receptor 12	NM_205823.2	127.2	263.7	131.7	306.6	107.3	132.8
Transthyretin	NM_013697.5	8198.6	12323.3	5331.2	11324.8	50.3	112.4
Ras-related associated with diabetes	NM_019662.2	699.4	1682.9	427.9	836.2	140.6	95.4
Transformation related protein 53 inducible nuclear protein 1	NM_021897.3 NM_001199105.1	917.6	1624.4	528.7	1016.2	77.0	92.2
Claudin 23	NM_027998.4	2564.6	3367.4	2346.5	4466.1	31.3	90.3

C, control mouse bladders; K, ketamine-injected mouse bladders.

**Table II:** Top ten downregulated genes with differential expression (fold change log 2 ≤ −1 and P<0.05) at 60-day ketamine treatment and statistical difference (P<0.05) at 30-day ketamine treatment in mouse bladders.

		Normalized intensity				Ratio of change (%)	
		
		30-day		60-day		(K-C)/C × 100%	
		
Gene name	Accession number	C	K	C	K	30-day	60-day
WAP four-disulfide core domain 3	NM_027961.1	102.6	46.6	528.3	59.7	−54.6	−88.7
Metallothionein 2	NM_008630.2	1230.5	782.7	5276.6	751.2	−36.4	−85.8
Tissue inhibitor of metallo-proteinase 1	NM_001044384.1 NM_011593.2	1445.2	551.3	3648.6	845.7	−61.9	−76.8
Solute carrier family 7, member 11	NM_011990.2	624.4	403.7	2992.5	708.8	−35.3	−76.3
Keratin 14	NM_016958.1	2972.3	1652.2	6385.7	1621.5	−44.4	−74.6
Glutamine fructose-6-phosphate transaminase 2	NM_013529.3	329.1	224.3	806.2	211.3	−31.8	−73.8
Macrophage scavenger receptor 1	NM_031195.2	457.3	283.3	891.6	281.0	−38.0	−68.5
Interleukin 33	NM_001164724.1 NM_133775.2	1403.7	705.9	3731.8	1207.9	−49.7	−67.6
C-type lectin domain family 4, member d	NM_00116316.1 NM_010819.4	151.6	73.6	201.6	66.5	−51.5	−67.0
Neuregulin 1	NM_178591.2	86.5	49.5	199.8	68.0	−42.8	−66.0
Keratin 78	NM_212487.4	241.5	166.6	857.4	365.9	−31.0	−57.3

C, control mouse bladders; K, ketamine-injected mouse bladders.

**Table III:** Top ten downregulated keratin genes following ketamine treatment for 60 days.

		Normalized intensity				Ratio of change (%)	
		
		30-day		60-day		(K-C)/C×100%	
		
Gene name	Accession number	C	K	C	K	30-day	60-day
Keratin 6a	NM_008476.3	42.6	24.3	985.4	46.0	−43.0	−95.3^a^
Keratin 13	NM_010662.1	130.9	170.0	1165.9	181.6	29.9	−84.4^a^
Keratin 14	NM_016958.1	2972.3	1652.2	6385.7	1621.5	−44.4^a^	−74.6^a^
Keratin 23	NM_033373.1	580.3	676.9	1926.2	722.6	16.6	−62.5^a^
Keratin 78	NM_212487.4	241.5	166.6	394.9	185.3	−31^a^	−57.3^a^
Keratin 5	NM_027011.2	6344.0	6327.0	9821.2	5129.2	−0.3	−47.8^a^
Keratin 6b	NM_010669.2	13.9	9.5	35.5	19.9	(NA)	−44
Keratin 8	NM_031170.2	13729.3	15504.3	20223.6	13109.0	12.9	−35.2^a^
Keratin 19	NM_008471.2	9891.2	7572.7	15246.7	10048.5	−23.4	−34.1^a^
Keratin 79	NM_146063.1	104.4	100.4	163.7	108.2	−3.9	−33.9^a^

a P<0.05, indicates a significant difference between the control and ketamine-injected groups. Rosetta Resolver^®^ was applied to detect signal noise for reducing the false positive, the signal was adjusted to NA if this sample was not qualified.

C, control mouse bladders; K, ketamine-injected mouse bladders.

**Table IV:** Four gene expression change data in normal saline and ketamine treatment for 30 and 60 days.

		Normalized intensity				Ratio of change (%)	
		
		30-day		60-day		(K-C)/C×100%	
		
Gene name	Accession number	C	K	C	K	30-day	60-day
Integrin α6	NM_008397.3	6661.3	9441.9	9028.3	7893.3	41.7^a^	−12.6
Integrin β4	NM_133663.2 NM_001005608.2	802.7	739.2	1082.0	394.7	−7.9	−63.5^a^
Claudin-1	NM_016674.4	1146.1	1002.7	3597.1	1209.6	−12.5	−66.4^a^
Keratin 20	NM_023256.1	473.0	568.4	1954.1	708.4	20.2	−63.7^a^

a P<0.05 between the control and ketamine-injected groups.

C, control mouse bladders; K, ketamine-injected mouse bladders.

## Abbreviations

FBS: fetal bovine serum
H&E: hematoxylin and eosin
PI: propidium iodide
RNase A: ribonuclease A
